# Microparticles Carrying Sonic Hedgehog Favor Neovascularization through the Activation of Nitric Oxide Pathway in Mice

**DOI:** 10.1371/journal.pone.0012688

**Published:** 2010-09-13

**Authors:** Tarek Benameur, Raffaella Soleti, Chiara Porro, Ramaroson Andriantsitohaina, Maria Carmen Martínez

**Affiliations:** 1 CNRS, UMR 6214, INSERM, U771, Université d'Angers, Faculté de Médecine, Angers, France; 2 Department of Biomedical Sciences, School of Medicine, University of Foggia, Foggia, Italy; University of Padova, Medical School, Italy

## Abstract

**Background:**

Microparticles (MPs) are vesicles released from plasma membrane upon cell activation and during apoptosis. Human T lymphocytes undergoing activation and apoptosis generate MPs bearing morphogen Shh (MPs^Shh+^) that are able to regulate *in vitro* angiogenesis.

**Methodology/Principal Findings:**

Here, we investigated the ability of MPs^Shh+^ to modulate neovascularization in a model of mouse hind limb ischemia. Mice were treated *in vivo* for 21 days with vehicle, MPs^Shh+^, MPs^Shh+^ plus cyclopamine or cyclopamine alone, an inhibitor of Shh signalling. Laser doppler analysis revealed that the recovery of the blood flow was 1.4 fold higher in MPs^Shh+^-treated mice than in controls, and this was associated with an activation of Shh pathway in muscles and an increase in NO production in both aorta and muscles. MPs^Shh+^-mediated effects on flow recovery and NO production were completely prevented when Shh signalling was inhibited by cyclopamine. In aorta, MPs^Shh+^ increased activation of eNOS/Akt pathway, and VEGF expression, being inhibited by cyclopamine. By contrast, in muscles, MPs^Shh+^ enhanced eNOS expression and phosphorylation and decreased caveolin-1 expression, but cyclopamine prevented only the effects of MPs^Shh+^ on eNOS pathway. Quantitative RT-PCR revealed that MPs^Shh+^ treatment increased FGF5, FGF2, VEGF A and C mRNA levels and decreased those of α5-integrin, FLT-4, HGF, IGF-1, KDR, MCP-1, MT1-MMP, MMP-2, TGFβ1, TGFβ2, TSP-1 and VCAM-1, in ischemic muscles.

**Conclusions/Significance:**

These findings suggest that MPs^Shh+^ may contribute to reparative neovascularization after ischemic injury by regulating NO pathway and genes involved in angiogenesis.

## Introduction

Microparticles (MPs) are small plasma membrane fragments shed by cells after blebbing due to activation and/or apoptosis. They play an important role in cell to cell communication because of their ability to act at distant site as well as locally, and to propagate the functional antigens of their parent cell [1].

It was documented that MPs are implicated in modulation of different stages of angiogenesis, although contradictory results have been reported in the literature. Indeed, the different responses evoked by MPs are dependent on their cellular origin, stimulus of their generation and their concentration. MPs shedding from endothelial cells (ECs) contain active proteases, able to promote matrix degradation, but also the machinery to generate them, presumably initiated by stimuli from environment. Thus, they induce proteolysis during cell migration and three-dimensional morphological organization during angiogenesis [2]. In contrast, another study showed that endothelial MPs are able to impair *in vitro* angiogenesis by affecting all parameters of the capillary network formation [3]. It has been shown that MPs released by apoptotic lymphocytes inhibited *in vitro* and *in vivo* angiogenesis, by enhancing ROS production, which leads suppression of vascular cell survival, proliferation and migration [4]. Furthermore, the same MPs are able to decrease NO production via PI3K [5]. On the other hand, MPs generated from human lymphocytes undergoing activation and apoptosis express morphogen Shh (MPs^Shh+^) at their surface and induce cell differentiation [6]. Besides, these MPs have concomitant effect of increasing NO production directly by Shh and PI3K pathways and decreasing ROS production by a mechanism dependent on PI3K and ERK cascades [7]. Moreover, we have observed that MPs^Shh+^ regulate multiple pathways related to *in vitro* angiogenesis, mainly through the production of pro-angiogenic factors and up-regulation of proteins involved in cell adhesion [8]. Thereby, the different effects evoked by MPs from apoptotic and activated/apoptotic lymphocytes are probably due to different stimulation at their origin and also to the absence and presence of Shh, respectively. Shh morphogen orchestrates several processes such as cell proliferation, differentiation and angiogenesis [9]. Concerning angiogenesis, it has been reported that activation of Shh cascade evoked *in vitro* capillary-like structures formation and *in vivo* new blood vessel generation [10]–[12]. Moreover, the effects promoted by Shh affect modulation of VEGF and eNOS activities [13] and involve PI3K/Akt pathway, which also belongs to intracellular mechanism for endothelial NO release. As reported above, we have shown that MPs^Shh+^ are able to differentially regulate cell events leading to *in vitro* angiogenesis [8]. To further validate the observed effects on *in vitro* angiogenesis, here we used a mouse model of hind limb ischemia, in order to investigate the efficacy of MPs^Shh+^ pro-angiogenic properties in neovascularization process with respect to recovery of blood flow, vascular density, implication of pathways which regulate NO production and expression of key factors involved in angiogenesis. Understanding the mechanism evoked by MPs in response to ischemia is essential for the development of new therapeutic strategies for ischemic cardiovascular diseases.

## Materials and Methods

### MP production

The human lymphoid CEM T cell line (ATCC, Manassas, VA) was used for MP production. Cells were seeded at 10^6^ cells/mL and cultured in serum-free X-VIVO 15 medium (Lonza, Walkersville, MD). MPs were produced as described previously [6]–[8]. Briefly, CEM cells were treated with phytohemagglutinin (5 µg/mL; Sigma-Aldrich, St. Louis, MO) for 72 h, then with phorbol-12-myristate-13-acetate (20 ng/mL, Sigma-Aldrich) and actinomycin D (0.5 µg/mL, Sigma-Aldrich) for 24 h. A supernatant was obtained by centrifugation at 750 *g* for 15 min, then at 1,500 *g* for 5 min to remove cells and large debris, respectively. MPs from the supernatant were washed after serial centrifugation steps (45 min at 14,000 *g*) and recovered in 400 µL NaCl (0.9% w/v). Washing medium from the last supernatant was used as control (vehicle). Determination of the amount of MPs was carried out by measuring total MP-associated proteins, using the Bradford method, and BSA (Sigma-Aldrich) for the standard curve, as previously described [6]–[8].

### Mouse Model of Hindlimb Ischemia

The University of Angers ethical committee approved the present protocol. All animal studies were carried out using approved institutional protocols and were conformed the *Guide for the Care and Use of Laboratory Animals* published by US National Institutes of Health (NIH Publication No. 85–23, revised 1996). Four groups of male Swiss mice (Charles River Laboratories, L'Arbresle, France), 6–8 weeks of age were treated, every three days for 21 days: (i) mice receiving i.v. injection of vehicle (control group, n = 15); (ii) mice receiving i.v. injection of MPs (10 µg/mL of blood) (n = 13); (iii) mice receiving i.v. injection of MPs after 30 min of i.p. injection of cyclopamine (10 mg/kg) (Biomol International, Plymouth Meeting, PA) (n = 7); (iv) mice receiving only i.p. injection of cyclopamine (n = 6). We have taken in consideration that total blood volume in the mouse is 6–8 ml of blood/100 g of body weight [14]. The animals were housed in a regulated environment with a constant ambient temperature of 24°C. They had free access to standard laboratory food and water.

Twenty four hours after the first injection, mice were anesthesized with isoflurane and underwent surgery to induce unilateral hindlimb ischemia. The ligation was performed on the left femoral artery proximal to the bifurcation to the saphenous and popliteal arteries as previously described [15], [16]. After 7 and 21 days of ligature, blood flow was measured as described below. At 21 day, mice were euthanized and tissues were sampled for biochemical and histological analysis. The procedure followed in the care and euthanasia of the study was in accordance with the European Community standards on the care and use of laboratory animals.

### Quantification of neovascularization: Laser-Doppler Blood Flow (LDBF) analysis

In order to provide a functional evidence of ischemia, laser doppler perfusion imaging was performed in anesthetized mice, as previously described [16]. Animals were settled on a heating plate to maintain a stable cutaneous temperature in order to minimize temperature variation throughout the experiments. Leg perfusion was then measured using a Laser Doppler flow probe (PF 408, Perimed, Stockholm, Sweden). Blood flow was recorded during ∼3 min. At least 2 flow measurements were performed per leg. Blood flow perfusion was expressed as a ratio of left (ischemic) to right (non-ischemic) leg, as described by Limbourg et al. [17].

### Vascular density

Vascular density, as an index of neovascularization, was examined by counting the number of vessels taken from the ischemic and non-ischemic limbs. Ischemic and non-ischemic gastrocnemius muscles were dissected and embedded in Tissue-Tek O.C.T (Sakura Finetek, Zoeterwoude, The Netherlands). Cryosections (7 µm) were fixed (5 min at −20°C) in 100% methanol, and saturated (1 h at room temperature) in blocking buffer (5% non fat dry milk in PBS and 0.05% Tween 20). Fixed and blocked tissue sections were incubated overnight at 4°C with rat anti-mouse CD31 antibody (BD Biosciences, San Jose, CA). After three washes, tissue sections were incubated (1 h at room temperature) with goat anti-rat IgG fluorescein-conjugated (Southern Biotech, Birmingham, AL) to identify vessels as described by Limbourg et al. [17]. After final washes, sections were mounted on glass slides. MRC-1024ES confocal equipment mounted on a Nikon Eclipse TE 300 inverted microscope was used for the optical sectioning of the tissue. Digital image recording was performed using the Laser Sharp Software. Vessels were quantified using ImageJ software and counted in at least four randomly selected fields for each muscle section, and the mean value for each section was calculated (magnification x40).

### NO determination by electronic paramagnetic resonance (EPR)

Detection of NO production was performed using a technique with Fe^2+^ diethyldithiocarbamate (DETC, Sigma) as spin trap. After 21 days of ligation, animals were euthanized, aorta and both ischemic and non-ischemic muscles were dissected and incubated for NO production for 30 min in Krebs-Hepes buffer containing: BSA (20.5 g/L), CaCl_2_ (3 mmol/L) and L-arginine (0.8 mmol/L). NaDETC (3.6 mg) and FeSO_4_.7H_2_O (2.25 mg) were separately dissolved under N_2_ gas bubbling in 10 mL volumes of ice-cold Krebs–Hepes buffer. These were rapidly mixed to obtain Fe(DETC)_2_ solution (0.4 mmol/L), which was used immediately to incubate tissues for 45 min at 37°C. Then, tissues were immediately frozen using liquid N_2_. NO measurement was performed using a table-top x-band spectrometer Miniscope (Magnettech, MS200, Berlin, Germany). Recording were made at 77°K using a Dewar flask. Instrument setting was 10 mW of microwave power, 1 mT of amplitude modulation frequency, 60 s of sweep time and 3 scans.

### Western blot analysis

In each experiment, the aorta and the skeletal muscle from both ischemic and non-ischemic leg were removed and frozen. Samples were homogenized (Polytron; PRO250, Monroe, CT) and lysed. Proteins were separated by SDS-PAGE and transferred onto nitrocellulose membranes. Membranes were then saturated at room temperature for 1 h in Tris buffer containing 1% Tween-20 and 5% BSA. Membranes were incubated overnight at 4°C with one of the following primary monoclonal antibodies: anti-human Shh, anti-human Ptc, anti-mouse Shh, anti-mouse Ptc (USBiological, Swampscott, MA), anti-endothelial NOS (eNOS), anti-caveolin-1 (BD Biosciences), anti-phospho-eNOS (Ser 1177), anti-Akt, anti-phospho-Akt (Ser 473) (Cell Signaling, Beverly, MA), and anti-VEGF (R&D systems, Minneapolis, MN). To visualize protein gel loading, a polyclonal rabbit anti-actin antibody (Sigma-Aldrich) was used at 1/2000 dilution. The membranes were then washed at least three times in Tris buffer containing 0.05% Tween-20 and incubated for 1 h at room temperature with the appropriate horseradish peroxidase (HRP)-conjugated secondary antibody (Amersham, Piscataway, NJ). The protein-antibody complexes were detected by ECL-Plus Chemiluminescence kit (Amersham) according to manufacturer's protocol.

### Quantitative real time RT-PCR analysis

In another set of experiments, the skeletal muscle from both ischemic and non-ischemic legs were frozen and used to investigate mRNA levels of 39 transcripts related to angiogenesis by quantitative RT-PCR (q-PCR). q-PCR analyses were carried out by Service Commun de Cytométrie et d'Analyses Nucléotidiques from Angers University, using a Chromo 4™ (Bio-Rad, Hercules, CA) and SYBR Green detection. Primers were designed using Primer3 software (http://frodo.wi.mit.edu/cgi-bin/primer3/primer3_www.cgi). Quantifications were realized according to the ΔCt method and the relative gene-expression levels were normalized using the geometric mean of three housekeeping genes as previously described [18]. Data were expressed as a ratio of ischemic on non-ischemic gene-level expression.

### Statistical analysis

Data are represented as mean ± SEM, *n* represents the number of mice. Statistical analyses were performed by Mann-Whitney U-tests (non-parametric). *P*<0.05 was considered to be statistically significant.

## Results

### Enhanced recovery of blood flow after induction of hindlimb ischemia in MPs^Shh+^-treated mice

**Figure 1:**
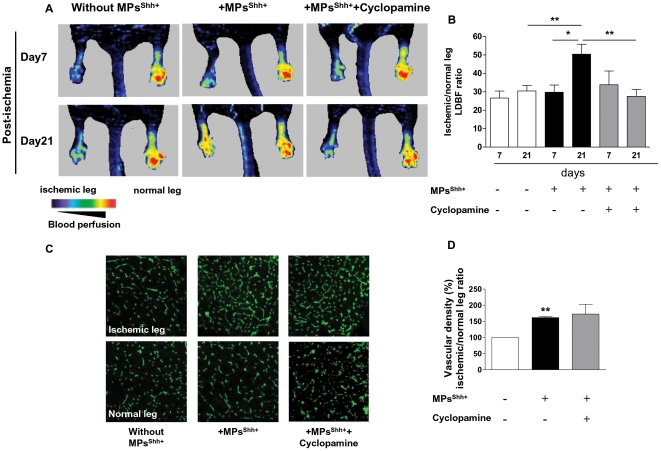
Quantitative evaluation of post-ischemic neovascularization after femoral artery ligature in mice. (A) Representative pseudocolour laser Doppler blood flow (LDBF) images at day 7 and 21 following ligation of the left femoral artery in mice receiving vehicle (without MPs^Shh+^) (n = 15), MPs^Shh+^ alone (n = 13), or with cyclopamine (n = 7). (B) Histogram showing the quantification of the limb perfusion as a ratio of blood flow reperfusion in ischemic and non-ischemic legs. (C) Representative immunofluorescence images of non-ligated (normal leg) and ischemic leg of gastrocnemius muscle sections stained with CD31 from mice receiving vehicle (without MPs^Shh+^), MPs^Shh+^, and MPs^Shh+^ with cyclopamine during 21 days. (D) Quantification of number of CD31-stained vessels of gastrocnemius muscle sections from mice receiving vehicle (n = 3), MPs^Shh+^ (n = 3), and MPs^Shh+^ with cyclopamine (n = 3). *P<0.05, **P<0.01. Data are expressed as ratio of ischemic to non-ischemic leg (mean ± SEM).

Serial blood flow measurements were performed sequentially in the ischemic (left) and non-ischemic (right) hind limbs and were analyzed by a LDBF image analyser and expressed as pseudocolour images (Figure 1A). Hindlimb ischemia was induced by ligating-excising the left femoral artery, and there was no significant difference in the degree of post-operative ischemia between groups (data not shown). Also, at day 7 after ligation, there was no difference in blood flow in all groups. However, 21 days after surgery, ischemic-to-non-ischemic limb perfusion ratio was modified and became evident in response to distinct treatments (Figure 1B). In control mice, the recovery of blood flow was not significantly different at day 7 and 21. In MPs^Shh+^-treated mice, the recovery of blood flow was significantly increased after 21 days from ligation compared to day 7 (*P*<0.05). Moreover, MPs^Shh+^-treated mice showed a significant increase of blood flow ratio (∼1.4 fold) compared with the control group (*P*<0.01). Interestingly, at day 21, treatment of mice with cyclopamine prevented the enhancement of blood flow perfusion evoked by MPs^Shh+^ (*P*<0.01). It should be noted that cyclopamine alone induced an increase of blood flow ratio when compared with control group (Figure S1A). Together, these findings suggest that MPs^Shh+^ are able to improve blood perfusion and this effect is directly mediated by Shh signalling, as illustrated by impaired blood flow recovery in the presence of Shh pathway inhibitor.

### MPs^Shh+^ enhance vascular density after induction of hind limb ischemia in mice

As illustrated in Figure 1C and 1D, after 21 days of ischemia, MPs^Shh+^ enhanced significantly the ratio of ischemic/non-ischemic vascular density in hind limbs by ∼62% compared with vehicle-treated mice. This observation is consistent with the findings that MPs^Shh+^ improve foot reperfusion. In contrast, cyclopamine failed to prevent the observed effects of MPs^Shh+^ on ischemic/non-ischemic vascular density ratio. Cyclopamine alone did not induce significant changes in ischemic/non-ischemic limb ratio of vessel number when compared to control group (Figure S1B).

### MPs^Shh+^ stimulate endogenous Shh expression on mouse ischemic muscle

**Figure 2:**
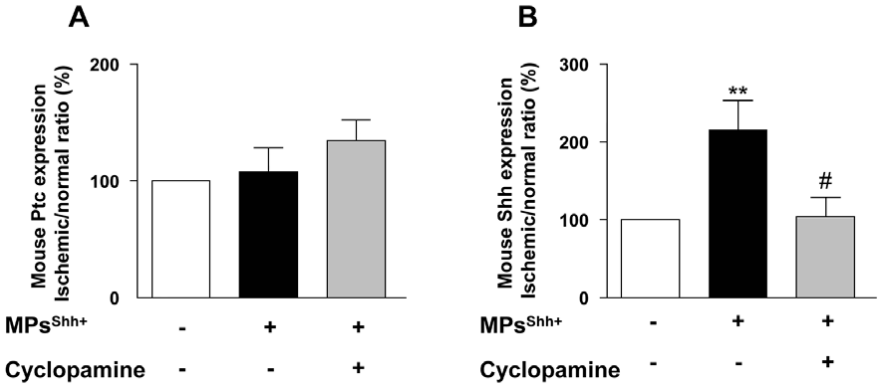
MPs^Shh+^ induce Shh expression in mouse skeletal muscles. Histograms showing the effects of MPs^Shh+^ on mouse Ptc (A) and Shh (B) expressions. Values are expressed as a ratio of ischemic/non-ischemic protein expression in arbitrary units (A.U.) as mean ± SEM (n = 5). **P<0.01 *vs* control group; #*P*<0.05 *vs* MPs^Shh+^-treated mice.

We have evaluated whether exogenous or endogenous Shh is involved in the effects of MPs^Shh+^. Neither human Shh nor Ptc were detected in skeletal muscles of mice after 21 days of MPs^Shh+^ treatment (not shown). Moreover, although mouse Ptc expression was not changed after MPs^Shh+^ injection, mouse Shh expression was enhanced in mice receiving MPs^Shh+^ (*P*<0.01) (Figure 2A and 2B). Furthermore, at day 21, treatment of mice with cyclopamine alone had no significant effect on both Ptc and Shh expressions (Figure S1C and S1D), but it prevented the enhancement of mouse Shh expression evoked by MPs^Shh+^ (*P*<0.05) (Figure 2A and 2B).

### MPs^Shh+^ stimulate NO production and enhanced VEGF expression in aorta

**Figure 3:**
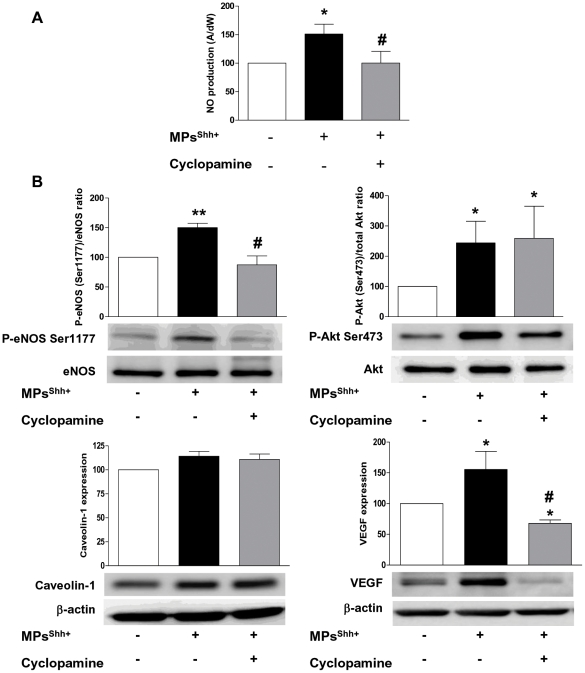
MPs^Shh+^ stimulate NO production and VEGF expression in mice aorta. (A) Quantification of the amplitude of the NO-Fe(DETC)_2_ complex signals in mice aorta. Values are expressed as amplitude/mg of dried weight of aorta in arbitrary units (mean ± SEM) (n = 3 to 6). (B) Histograms show the ratio of phosphorylation of eNOS Ser 1177 *vs* total eNOS, phosphorylation of Akt *vs* total Akt, and caveolin-1 and VEGF A expressions. Immunoblots were quantified by densitometric analysis and normalized with either the full form of corresponding protein or with β-actin (for caveolin-1 and VEGF A). Values are expressed in arbitrary units (A.U.) as mean ± SEM (n = 4). *P<0.05, **P<0.01 *vs* control group; #*P*<0.05 *vs* MPs^Shh+^-treated mice.

To determine whether MPs^Shh+^ effects are not restricted to ischemic area, but may target other vascular beds, we used aorta as control. Firstly, we evaluated NO production in aorta. Aortic rings from mice treated with either vehicle, MPs^Shh+^ alone or together with cyclopamine, preincubated with Fe(DETC)_2,_ exhibited an EPR feature of signal derived from NO-Fe(DETC)_2_ (data not shown). As shown in Figure 3A, MPs^Shh+^ enhanced significantly NO production in aorta compared to control (*P*<0.05). Moreover, whereas cyclopamine alone did not modify NO production (Figure 1SE), treatment with cyclopamine prevented the increase of NO induced by MPs^Shh+^ (*P*<0.05). Next, we investigated the pathway involved in MPs^Shh+^-induced NO production. MPs^Shh+^ treatment significantly enhanced phosphorylation of eNOS and Akt on their activator sites (Ser 1177 and Ser 473, respectively), without affecting their expressions (Figure 3B). Interestingly, blockade of Shh cascade by cyclopamine suppressed the MPs^Shh+^-induced activation of eNOS, but not that of Akt. However, MPs^Shh+^ treatment, as well as the administration of cyclopamine, did not modify caveolin-1 expression. Moreover, because VEGF plays a role in NO regulation, and VEGF-induced angiogenesis is mediated by NO, we examined its expression. MPs^Shh+^ significantly promoted an increase of VEGF expression (*P*<0.05) being inhibited by cyclopamine administration.

### MPs^Shh+^ enhance NO production and eNOS expression in skeletal muscles

**Figure 4:**
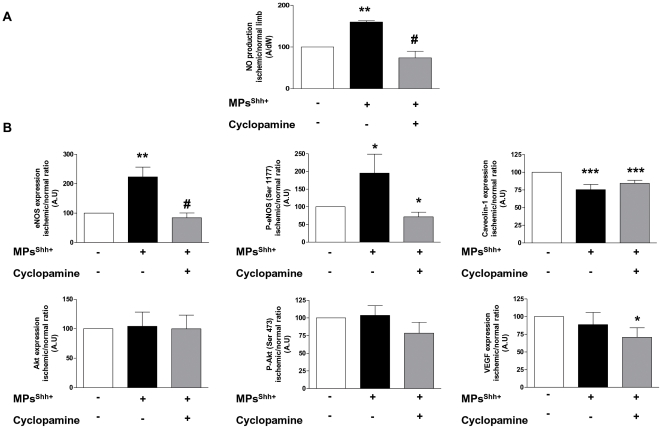
MPs^Shh+^ activate eNOS pathway in skeletal muscles. (A) Quantification of the amplitude of the NO-Fe(DETC)_2_ complex signals in muscles. Values are expressed as amplitude/mg of dried weight of skeletal muscles in arbitrary units (mean ± SEM) (n = 4 to 6). (B) Histograms showing the effects of MPs^Shh+^ on eNOS expression and phosphorylation on Ser 1177, caveolin-1 expression, Akt expression and phosphorylation on Ser 473 and VEGF A expression. Values are expressed as a ratio of ischemic/non-ischemic protein expression in arbitrary units (A.U.) as mean ± SEM (n = 4 to 9). *P<0.05, **P<0.01, ***P<0.001 *vs* control group; #*P*<0.05 *vs* MPs^Shh+^-treated mice.

Consistent with the data obtained on aorta, skeletal muscles from MPs^Shh+^-treated mice exhibited a significant increase of NO production (*P*<0.01) (Figure 4A). This effect was entirely reversed after cyclopamine administration, indicating that MP-induced NO release is sensitive to inhibition of Shh signaling (Figure 4A). It should be noted that cyclopamine alone did not modify NO production in skeletal muscles (Figure S1C).

MPs^Shh+^ administration induced a significant increase in eNOS expression (*P*<0.05), and its activation as shown by the eNOS phosphorylation at Ser 1177 (Figure 4B). Moreover, expression of caveolin-1, which plays a key role in negative regulation of eNOS, was decreased in muscles from MPs^Shh+^-treated mice (*P*<0.001). In contrast, neither expression nor activation of Akt was influenced by MPs^Shh+^ administration. Also, VEGF expression was not affected by MPs^Shh+^. When Shh cascade was pharmacologically inhibited, the effect induced by MPs^Shh+^ on eNOS expression was completely prevented, as well as its activation; whereas caveolin-1 expression was not affected compared to MPs^Shh+^-treated mice. Concerning VEGF, cyclopamine was able to significantly decrease its expression. On the other hand, cyclopamine alone did not affect expression levels of eNOS and VEGF (Figure S1F and S1G).

### MPs^Shh+^ regulate gene expression of angiogenic factors in skeletal muscles

To investigate candidate targets of MPs^Shh+^ implicated in neovessels formation process, we have evaluated the mRNA levels of 39 different angiogenic factors by q-PCR in skeletal muscles (Table 1). MPs^Shh+^ were able to enhance FGF2, FGF5 and VEGF A, B and C mRNA levels, by a Shh-dependent mechanism for FGF5 and VEGF A and by a Shh-independent mechanism for FGF2 and VEGF B and C (Table 1). In addition, MPs^Shh+^ decreased those of FLT-4, KDR, MMP-2, MT1-MMP and TSP-1, that were reversed when Shh signalling was blocked (Table 1). Also, MPs^Shh+^ decreased expression of α5-integrin, E-selectin, HGF, IGF-1, MCP-1, TGFβ1, TGFβ2 and VCAM-1 independently of Shh pathway (Table 1).

**Table 1:** Effects of MPs^Shh+^ on different mRNA expression on ischemic muscles.

mRNA	Ratio(MPs *vs* CTL)	*P* value(MPs *vs* CTL)	Ratio(MPs+Cycl vs MPs)	*P* value(MPs+Cycl *vs* MPs)
**α5-integrin**	**0.52**	**<0.05**	**1.03**	**ns**
Ang-1	0.83	ns	1.87	ns
Ang-2	1.2	ns	0.80	ns
β3-integrin	1.06	ns	1.49	ns
**E-selectin**	**0.29**	**<0.05**	**2.03**	**ns**
FGF1	1.21	ns	2.43	ns
**FGF2**	**1.43**	***<*** **0.01**	**1.06**	**ns**
FGF3	ND		ND	
**FGF5**	**3.83**	**<0.05**	**0.21**	**<0.05**
FGF7	0.88	ns	0.90	ns
FGF8	ND		ND	
FGF10	0.92	ns	2.32	ns
FGFR1	0.97	ns	1.18	ns
FGFR2	0.88	ns	0.32	ns
FLT-1	ND		ND	
**FLT-4**	**0.48**	**<0.05**	**3.41**	**<0.05**
**HGF**	**0.29**	**<0.05**	**1.00**	**ns**
ICAM-1	0.73	ns	1.80	ns
**IGF-1**	**0.30**	**<0.05**	**1.02**	**ns**
IL-1β	ND		ND	
IL-6	ND		ND	
**KDR**	**0.64**	**<0.05**	**1.78**	**<0.05**
**MCP-1**	**0.38**	**<0.05**	**1.29**	**ns**
**MMP2**	**0.38**	***<*** **0.01**	**2.05**	**<0.05**
**MT-1MMP**	**0.43**	***<*** **0.01**	**2.05**	**<0.05**
PECAM-1	1.01	ns	1.09	ns
SDF-1	0.67	ns	0.94	ns
**TGFβ1**	**0.58**	**<0.05**	**1.27**	**ns**
**TGFβ2**	**0.72**	**<0.05**	**1.31**	**ns**
Tie1	0.84	ns	1.67	ns
Tie2	0.88	ns	1.61	ns
TNFα	ND		ND	
**TSP-1**	**0.14**	***<*** **0.01**	**2.91**	**<0.05**
**VCAM-1**	**0.68**	**<0.05**	**1.69**	**ns**
VE-cadherin	0.97	ns	0.95	ns
**VEGF A**	**1.95**	**<0.05**	**0.60**	**<0.05**
**VEGF B**	**1.61**	**<0.05**	**1.17**	**ns**
**VEGF C**	**1.32**	**<0.05**	**1.01**	**ns**
VEGF D	1.18	ns	0.75	ns

Value are expressed as ratio of mRNA expression ischemic/normal muscle from MPs^Shh+^-treated mice versus control mice and from MPs^Shh+^ + Cycl- versus MPs^Shh+^-treated mice (n = 3 to 6). ND = No detected.

## Discussion

The present study is the first report showing the stimulatory effects of MPs^Shh+^ on *in vivo* neovascularization in post-ischemic context and the resultant blood flow recovery in an experimental model of hind limb ischemia. MPs^Shh+^ treatment induces an increase on Shh expression in ischemic skeletal muscle that was prevented by the Shh inhibitor, cyclopamine. The effects evoked by MPs^Shh+^ involve NO pathway and differential modulation of angiogenic factor expressions. However, not all the events implicated on neovascularization and triggered by MPs^Shh+^ are modified in the presence of cyclopamine, indicating that either some of the mechanisms involved are independent of Smo receptor activation or other molecules carried by MPs play a role in this phenomenon.

The formation of new blood capillaries is an important component of pathological tissue repair in response to ischemia. The development of an effective collateralization in the ischemic zone involves angiogenesis, which is the sprouting of new capillaries, and arteriogenesis, the development of arterial structures from small preexisting collateral vessels [19]. The present study demonstrates that MPs^Shh+^ treatment stimulates blood flow recovery in ischemic limbs after femoral artery ligation. This effect is mediated directly by Shh as illustrated by the abrogation of the capacity of MPs^Shh+^ in increasing perfusion in the presence of Shh antagonist, cyclopamine. In addition, expression of endogenous Shh was enhanced after 21 days of treatment with of MPs^Shh+^, and this effect was prevented in the presence of cyclopamine. These findings corroborate those obtained by Pola et al. [20] showing that Shh has a regulator role on angiogenesis during muscle regeneration after ischemia. Although we cannot exclude an increase in NO-mediated vasodilation induced by MPs^Shh+^ (see below), it was demonstrated that the stimulation of blood flow recovery was accompanied by an increase in vascular density in ischemic hind limb muscles suggesting that MPs^Shh+^-induced stimulation of blood flow reperfusion is due to neovessel formation. In contrast, Shh pathway inhibition did not prevent MPs^Shh+^-induced increase in vascular density in skeletal muscles. It is plausible that, in the presence of cyclopamine, non-functional vessels are formed as suggested by the fact that vessels are present under these conditions, but no flow recovery was detected.

Although a large number of studies highlight the role of Shh in angiogenesis, data reported in the literature are contradictory depending on the inhibition of either exogenous or endogenous Shh. Indeed, Shh seems to exert a dualistic action in cardiac ischemia in which high exogenous levels are able to foster tissue repair and endogenous Shh seems to be deleterious [21]. In the present study, we have observed that of MPs^Shh+^ treatment did not affect expression levels of both human (exogenous) Shh and Ptc proteins; however, MPs^Shh+^ treatment was able to induce mouse (endogenous) Shh protein expression when compared to control mice. These data suggest that after 21 days, MPs^Shh+^ treatment activates endogenous Shh pathway which seems to be beneficial in ischemic muscles as suggested by Pola et al [20]. In addition, although cyclopamine alone increased blood flow recovery at 21 days, this was not accompanied with changes in vascular density, NO production, and expression or Ptc, Shh, eNOS and VEGF suggesting that the cyclopamine effects on basal Shh pathway are minimal.

It has been shown that Shh treatment induces an increase on NO production via PI3kinase/Akt pathway in human ECs [22]. In the present study, MPs^Shh+^ treatment was able to promote NO production in non-ischemic area (aorta). In addition, in non-ischemic area, MPs^Shh+^ treatment activates both eNOS and Akt pathways as reported in our previous study [7]. Indeed, MPs^Shh+^ have been described able to activate the phosphatidylinositol 3-kinase (PI3K)/Akt pathways leading to rapid and sustained activation and enhanced expression of eNOS in ECs, aorta and lungs [7]. Furthermore, the effects on eNOS activation were sensitive to cyclopamine accordingly with our previous data [7]. In the present study, we also found that, in aorta, MPs^Shh+^ increased expression of VEGF by a mechanism sensitive to cyclopamine, in accordance with the effects evoked by MPs^Shh+^ in ECs [8]. VEGF, in addition to be pivotal angiogenic factor, plays an essential role in NO regulation. Of note is that exogenous Shh protein has been reported to induce eNOS and VEGF expressions in rat corpora cavernosa [13]. Thus, Shh may act as a coordinator of crosstalk between VEGF and NO and that eNOS and VEGF display synergism in promoting NO production.

Regarding skeletal muscles, MPs^Shh+^ induced an increase in NO production through the activation of the Shh pathway. This was associated with enhanced eNOS expression and its phosphorylation on the activator site. Concomitantly MPs^Shh+^ decreased caveolin-1 expression, which acts as negative regulator of eNOS activity and NO production [23], but this occurs independently of activation of Shh pathway. On the other hand, MPs^Shh+^ did affect neither Akt expression nor activation, indicating that, in skeletal muscles, eNOS activation induced by MPs^Shh+^ occurred by an Akt-independent mechanism. In this way, it has been reported that eNOS can be activated through a protein kinase C (PKC)-sensitive, but PI3K/Akt-independent pathway [24], [25]. Further experiments are necessary to determine the kinase implicated in the phosphorylation of eNOS evoked by MPs^Shh+^. It should be noted that, the fact that, in the presence of MPs^Shh+^ plus cyclopamine, eNOS phosphorylation and VEGF A expression decreased to lower levels than basal levels. It might be possible that the activation by MPs^Shh+^ is needed to potentiate the inhibitory effect of cyclopamine that cannot be observed under basal conditions. Moreover, the above effects might not be linked to the Shh pathway inhibition since cyclopamine alone had no effect on these proteins (Figure S1F and 1G). We cannot distinguish about the two hypotheses, nevertheless, the basal conclusion that MPs^Shh+^ induced an increase in NO production through the activation of the Shh pathway still holds.

Moreover, our results suggest that MPs^Shh+^ can modulate new vessels formation either at transcriptional, and at post-transcriptional level. Indeed, MPs^Shh+^ strongly influenced the angiogenic switch as revealed by the analysis of transcripts of a large number of genes involved in angiogenesis. For example, they enhanced expression of important angiogenic growth factors such as FGF2, FGF5 and VEGF (A, B and C). Accordingly with the present data, Giordano and coworkers [26] have described that intracoronary gene transfer of FGF5 increases blood flow in an ischemic region of the heart. Also, FGF2 induced stable collateral growth in ischemic hind limb [27]. Moreover, it has been reported that VEGF(s) expression in skeletal muscles results in the generation of new blood vessels [28]. Surprisingly, the enhanced mRNA expression of VEGF induced by MPs^Shh+^ treatment was not accompanied the corresponding increase of VEGF protein levels. Several hypotheses can explain these results. On one hand, it is plausible that expression of VEGF is delayed when compared to its mRNA expression. On the other hand, it is possible that the increase of translation to VEGF protein in ischemic/normal muscles is performed at low levels which may be below the threshold of detection for the antibody used in the present study. Similar findings have been previously described in astrocytes by others [29] and by our group [8]. Furthermore, MPs^Shh+^ decreased expression of the potent antiangiogenic protein, TSP-1. Consistent with the present study, Ridnour and collaborators [30] have demonstrated that NO-induced angiogenesis is regulated by the ability of NO to down-regulate TSP-1 expression.

Surprisingly, while mRNA levels of VEGFs were increased by MPs^Shh+^ treatment, expression of its receptors, FLT-4 and KDR, was decreased. It is likely that a down-regulation of expression of both types of receptors by MPs^Shh+^ treatment or that expression of VEGF receptors is delayed when compared to mRNA expression of their ligands, as previously described in human ECs [8]. Nevertheless, several pro-angiogenic factors are over-expressed, whereas anti-angiogenic factors are down-regulated suggesting that MPs^Shh+^ could induce angiogenesis through modulation of the balance between pro- and anti-angiogenic factor expressions. Furthermore, all effects of MPs^Shh+^ treatment in mRNA level expression were partially or totally reversed by inhibition of Shh signalling, suggesting that a direct stimulation of vascular cells by Shh associated with MPs is mandatory to favour formation of neo-vessels.

In addition, MPs^Shh+^ treatment decreased MT1-MMP and MMP-2 expression levels by a Shh-dependent mechanism. These findings may be explained by the fact that MMPs expressions and activities are temporally regulated [31], acting mainly in the very early phases of the initiation of angiogenesis. In fact, there are a number of evidences showing that MMP-1 is present at the leading tip of invading cells [32], [33], and by its ability to activate MMP-2, extends its degrading effect on extracellular matrix [32], [34]. Once neo-vessels are formed, the interaction between pericytes and the newly formed endothelial tubes is accompanied by silencing of MMPs activities which contributes to vascular stabilization [35]. All these regulated events may explain the decrease of their expression after MPs^Shh+^ treatment, at a stage at which neovascularization has already occurred.

The remained question is by which mechanism(s) MPs^Shh+^ are able to induce endogenous Shh expression. It has been shown that NO improves the effect of the ischemia induced in liver, kidney and testicular tissue as measured by damage markers and concomitantly increased Shh expression indicating that NO acts via increase of Shh expression in ischemic tissue [36]–[38]. A similar mechanism might occur in the present model. Indeed, MPs^Shh+^ evoke NO production that in its turn could activate endogenous Shh expression.

### Limitations of the study

We have previously shown that to generate MPs carrying Shh from lymphocyte T cells, concomitant treatment with PHA + PMA + Actinomycin D is needed [6]. Although the use of chemicals in the culture to induce Shh expression could be limited in order to avoid collateral/deleterious effects of MPs, it is clear that MPs carrying Shh might represent a potential therapeutic tool in pathologies associated with failed angiogenesis.

These data collectively show that MPs harbouring Shh may contribute to blood vessel growth, maturation and stabilization in a vascular network by reciprocally regulating the pro- and anti-angiogenic factors through the increase of NO production, under post-ischemic conditions. Thus, the present study highlights the potential usefulness of MPs^Shh+^ for therapeutic angiogenesis in pathologies associated with peripheral ischemia.

## Supporting Information

Figure S1 Effects of treatment of cyclopamine alone. (A) Histograms showing the quantification of the limb perfusion at day 7 and 21, as ratio of blood flow reperfusion in ischemic and non-ischemic legs in control (CTL) (n = 3) and cyclopamine (CYCL)-treated group (n = 3). *P<0.05 vs. CTL (B) Quantification of number of CD31-stained vessels of gastrocnemius muscle sections from mice receiving vehicle (CTL) or cyclopamine (CYCL) alone. Data are expressed as ratio of ischemic to non-ischemic leg (mean ± SEM) (n = 3). (C, D) Histograms showing the effects of cyclopamine (CYCL) on Ptc (C) and Shh (D) expressions. Values are expressed as a ratio of ischemic/non-ischemic protein expression in arbitrary units (A.U.) as mean ± SEM (n = 3). (E) Quantification of the amplitude of the NO-Fe(DETC)2 complex signals in muscles from control (CTL ) or cyclopamine (CYCL)-treated mice. Values are expressed as amplitude/mg of dried weight of skeletal muscles in arbitrary units (mean ± SEM) (n = 3). (F, G) Histograms showing the effects of cyclopamine (CYCL) on eNOS (F) and VEGF A (G) expressions. Values are expressed as a ratio of ischemic/non-ischemic protein expression in arbitrary units (A.U.) as mean ± SEM (n = 3).
(0.76 MB TIF)
