# Supplementary figures and images for: Microparticles Carrying Sonic Hedgehog Favor Neovascularization through the Activation of Nitric Oxide Pathway in Mice
Source: PLoS One. 2010 Sep 13;5(9):e12688. doi: 10.1371/journal.pone.0012688 (PMC2938335; doi:10.1371/journal.pone.0012688)

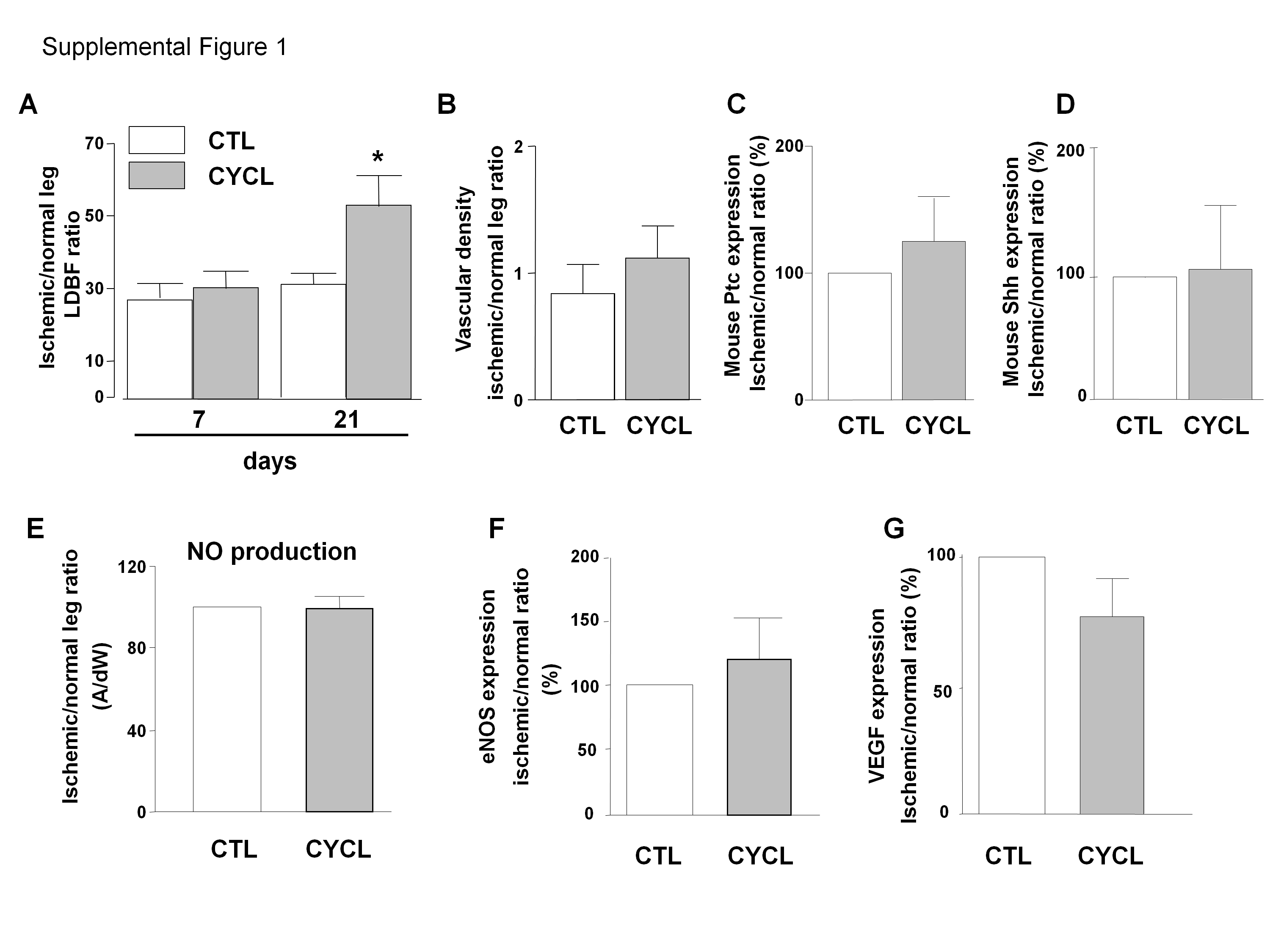

Supplement: Figure S1 — Effects of treatment of cyclopamine alone. (A) Histograms showing the quantification of the limb perfusion at day 7 and 21, as ratio of blood flow reperfusion in ischemic and non-ischemic legs in control (CTL) (n = 3) and cyclopamine (CYCL)-treated group (n = 3). *P<0.05 vs. CTL (B) Quantification of number of CD31-stained vessels of gastrocnemius muscle sections from mice receiving vehicle (CTL) or cyclopamine (CYCL) alone. Data are expressed as ratio of ischemic to non-ischemic leg (mean ± SEM) (n = 3). (C, D) Histograms showing the effects of cyclopamine (CYCL) on Ptc (C) and Shh (D) expressions. Values are expressed as a ratio of ischemic/non-ischemic protein expression in arbitrary units (A.U.) as mean ± SEM (n = 3). (E) Quantification of the amplitude of the NO-Fe(DETC)2 complex signals in muscles from control (CTL ) or cyclopamine (CYCL)-treated mice. Values are expressed as amplitude/mg of dried weight of skeletal muscles in arbitrary units (mean ± SEM) (n = 3). (F, G) Histograms showing the effects of cyclopamine (CYCL) on eNOS (F) and VEGF A (G) expressions. Values are expressed as a ratio of ischemic/non-ischemic protein expression in arbitrary units (A.U.) as mean ± SEM (n = 3). (0.76 MB TIF) [file pone.0012688.s001.tif]
